# Evaluation of Morphological Variations of External Ear between the Nepalese and Indian Students of A Medical College

**DOI:** 10.31729/jnma.3912

**Published:** 2018-12-31

**Authors:** Sarbada Makaju, Sonam Chaudhary, Karnaklata Iyer

**Affiliations:** 1 Department of Anatomy, Manipal College of Medical Sciences, Pokhara, Nepal; 2 Department of Physiology, Manipal College of Medical Sciences, Pokhara, Nepal

**Keywords:** *biometrics*, *external ear*, *prosthesis*, *shape of the ear*

## Abstract

**Introduction:**

The external ear is made up of the elastic cartilage. It is considered constant from the birth till death. Therefore, it's morphological variation plays important role in forensic medicine. The objective of this study was to identify this morphological variation among different nationality and the gender.

**Methods:**

The study was conducted among 50 Nepali students and 50 Indian students of Manipal College of Medical Sciences. The simple random sampling was done. The morphological variation of external ear including different nature of shape of tragus, anti-tragus, lobe and margin of helix were studied on different gender and nationalities.

**Results:**

The highest distribution in shape of tragus was round in Nepali 42 (84%) and Indian 37 (74%). Most of Nepali students had flat 26 (52%) shape of anti-tragus and medium for Indian 25 (50%). The maximum ear attached lobe were found in both Nepali and Indian 27 (54%) respectively. Most of the Indian students had elongated shape of helix 24 (48%) whereas in Nepalese round shape of helix 31 (62%). The male participants had maximum round shape of tragus 42 (76.3%), flat shape of anti-tragus 28 (50.9%), free ear lobe 28 (50.9%) and shape of the margin of helix were round 29 (52.7%). The female participants had maximum round shape of tragus 37 (82.2%), medium shape of anti-tragus 22 (48.8%), attached ear lobe 27 (60%), and round shape of margin of helix 25 (55.56%).

**Conclusions:**

The morphology of the external ear varies with each individual. However, it shows it couldn't differentiate their nationalities and gender.

## INTRODUCTION

The anatomy of the external ear is considered as the most prominent and defining structure of the face. It has irregular surface. The external ear consists of the elastic cartilage with the properties of elasticity except the lobes of the ear. Genetically, the shape of it is considered as one of the dominate character.^1^ The authors came with theory that the shape of the external ear is fixed and it doesn't shows any changes from birth till death.^2^

It also shows variation according to the sex and ethnicity whereas in the world of forensic medicine it is considered as one of the aid for sex determination.^3–4^ But in Chinese, the prominent ear significance as positive sign in life span.^5^ Similarly, in Nepal it also denotes the sign of longevity. For the surgeon, knowing the variation of it will help in reproducing a correct anatomical ear.^6–8^

Therefore, the aim of this study is to compare the morphological variation of external ear between nationalities and gender.

## METHODS

The descriptive cross-sectional study was carried out in Manipal College of Medical Sciences in between July 2017 to March 2018 after getting the ethical clearance from the Institutional Review Committee-MEMG-IRC Ref-1296. A total of 100 subjects were enrolled in the study with equal distribution among Nepalese and Indians students of Manipal College of Medical Sciences. The sample size was calculated based on the prevalence of Nepali Students in Manipal college of Medical Sciences which is 60%.


$$\text{Sample size (N) =}{\text{Z}}^{2}\times {\text{P(1-P)/e}}^{2}$$

where P = 0.6 and z = the confidence interval of 90%

Sample size for this study is 64.

The participations were included after taking verbal informed consent among the students between 1830 years of age by a simple random sampling method. Any history of surgery, trauma and congenital disorder of ear were excluded. The features of ear considered were shape of anti-tragus, shape of tragus, lobe and margin of helix. The following different categories in the selected features were descriptive analysed in SPSS version 20.

1. Shape of anti-tragus
  - Flat anti-tragus
  - Medium anti-tragus
  - Prominent Anti-tragus
2. Shape of tragus
  - Knob shaped Tragus
  - Long shaped tragus
  - Round shaped
3. Lobe
  - Attached lobe
  - Free lobe
4. Margin of helix
  - Elongated
  - Round
  - Narrow
  - Angulated

## RESULTS

**Figure 1.**
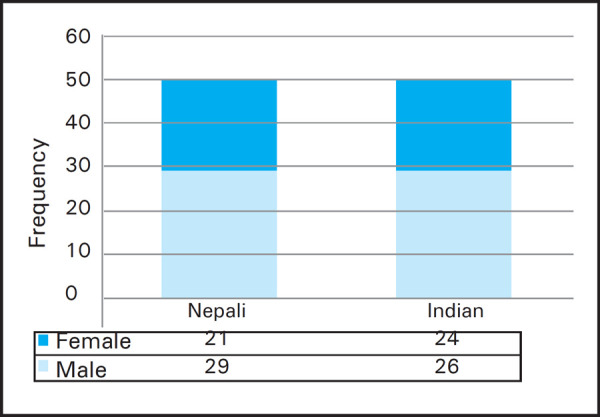
Distribution of total participants as per gender and nationality.

The male participants were comparatively more among Nepali in comparison to Indian (Figure 1).

**Figure 2.**
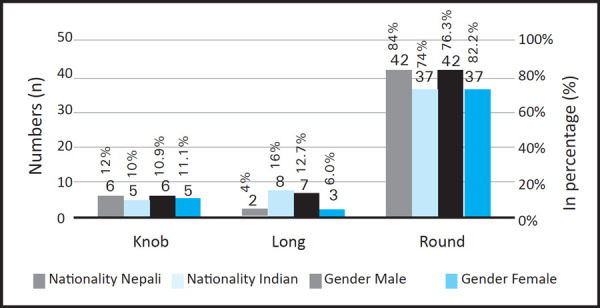
Variation of shape of tragus according to the nationality and gender (n=100).

Round shape of tragus was common in both the nationality and gender (Figure 2).

**Figure 3.**
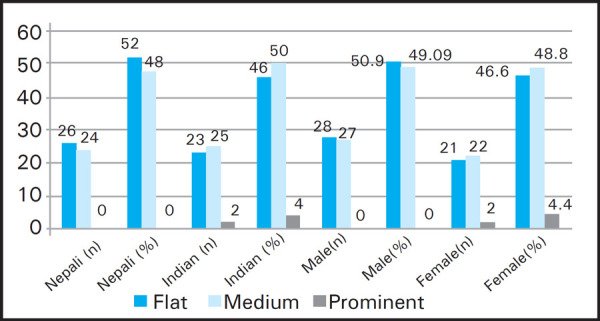
Variation of shape of anti-tragus according to the nationality and gender (n=100).

Nepalese participants had maximum flat shape of anti-tragus where as it was medium shape in case of Indians. Similarly, male had maximum flat shape of anti-tragus whereas it was medium shape for females (Figure 3).

**Figure 4.**
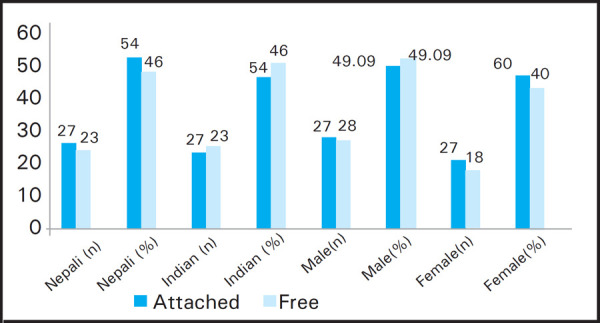
Variation in shape of lobe according to the nationality and gender (n=100).

Maximum participants of both nationalities had attached ear lobe. The male participants had maximum free ear lobe whereas it was attached in case of females (Figure 4).

**Figure 5.**
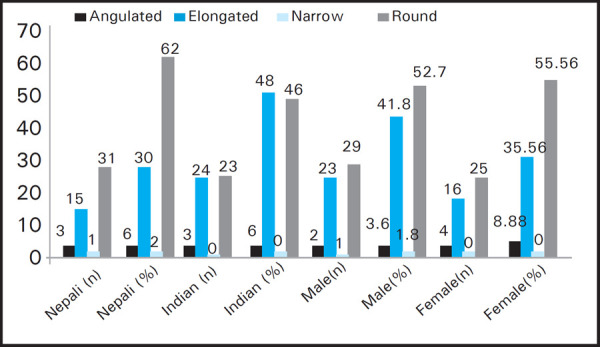
Variation in shape of margin of helix according to the nationality and gender (n=100).

Round shape margin of the helix was more in Nepali whereas in Indian elongated shape of helix was present (Figure 5).

## DISCUSSION

The shape of the external ear plays very important role in various aspects. This study which consisted of 50 Nepali and 50 Indian students from both gender found very less differences in the morphological distribution of the external ear. The round shape of tragus was common in both the nationalities and the gender. Whereas, the least common was long shape of tragus among Nepali 2 (4%) and knob shape in Indian 5 (10%). But in case of gender, the least common shape of tragus of male and female are knob and long shaped respectively. However, the shape of the anti-tragus was flat and medium shape was common in Nepali (52%) and Indian (50%) respectively. In comparison between male and female, male participants also had more flat shape of anti-tragus (50.9%) and female had medium shape (48.8%). The prominent shape of anti-tragus was least to be found among participants.

In this study attached ear lobes were common in both the nationality showing 54% but in case of the gender, females (60%) are having more attached lobe. This finding is in consistent with the Indian study which found maximum numbers were attached ear lobes (65%).^9^ Whereas, in Iraqi population, the free ear lobe were more common in female than in male.^10^ Whereas, the study conducted among the male Indian Rickshaw drivers shows 65.14% free and 34.85% attached lobe.^11^

The common shape of margin of helix was round in Nepali (62%) and elongated in Indian (48%). It is found that the distribution of narrow shape of margin of helix was least in both the nationalities. Whereas, in gender the round shape of margin of helix was most common among male (52.7%) and female (55.56%). However, this finding is contrast with the North Indian population with minimum distribution of round shape of margin of helix.^12^

This study resulted that the variation of external ear features is not specific among particular gender or nationality considered. This result is consistent with the finding of Cameriere R et al.^13^ Finally, the data represented in the study would have provided more reliable result with larger scale of sample. However, despite of this limitation it is valuable for the prosthetic surgery. Similarly, lack of uniformity in distribution of external ear features shows the individual variations which exist that can be considered as a personal identity marker. This can also be used as a method of biometrics which is cheaper and easy than the other aids.^11^

## CONCLUSIONS

The shape of the external ear features varies in each individual as it is carried genetically. Whereas, the comparison of frequency distribution pattern in the morphology of these external ear features shows minimum differences between the nationality and gender.
